# Long-Term Bladder Dysfunction and Bilateral Obstructive Megaureter in VACTERL Syndrome: A Case Report of Challenging Urological Management

**DOI:** 10.3390/reports8040239

**Published:** 2025-11-19

**Authors:** Maria Escolino, Paolo Caione, Claudia Di Mento, Mauro Porcaro, Ciro Esposito

**Affiliations:** 1 Pediatric Surgery Unit, Federico II University Hospital, 80131 Naples, Italy; 2 Pediatric Urology Unit, Salvator Mundi International Hospital, 00152 Rome, Italy; 3 Pediatric Radiology Unit, Federico II University Hospital, 80131 Naples, Italy

**Keywords:** VACTERL, bladder dysfunction, megaureter, vesicoureteral reflux, obstruction

## Abstract

**Background and Clinical Significance**: VACTERL association is a rare spectrum of congenital malformations that may involve the genitourinary system. We describe a challenging case of hypotonic, hyporeflexic, large-capacity bladder with bilateral obstructive megaureter in a boy with VACTERL syndrome, highlighting diagnostic and therapeutic challenges. **Case Presentation**: A 16-year-old boy with VACTERL syndrome, previously operated for esophageal atresia, Fallot’s tetralogy, Y-type urethral duplication, and bilateral vesicoureteral reflux, presented with breakthrough urinary tract infections, orchiepididymitis, and flank pain. Investigations revealed an enlarged bladder capacity (1000 mL), detrusor underactivity, high post-void residual volume, and bilateral hydronephrosis with megaureter. Obstruction of the bladder neck and neurological causes were excluded. After multidisciplinary discussion, bilateral ureteral reimplantation and limited reductive cystoplasty were performed. Histology revealed granulomatous foreign-body reaction due to previous bulking agent injection. Postoperative course was uneventful. At the three-year follow-up, the patient is asymptomatic with normal voiding and preserved renal function. **Conclusions**: This case illustrates the diagnostic and therapeutic challenges of managing late urological complications in a VACTERL patient with pre-existing urinary anomalies. The overlap of congenital and iatrogenic factors made the diagnostic pathway complex, requiring careful exclusion of neurogenic and mechanical causes. A tailored surgical strategy restored bladder function and preserved renal outcome.

## 1. Introduction and Clinical Significance

VACTERL association is defined by the presence of at least three of the following anomalies: vertebral defects, anorectal malformations, cardiac anomalies, tracheoesophageal fistula, renal anomalies, and limb defects [1]. Its estimated incidence is about 1 in 10,000 live births [2]. Renal and urinary tract anomalies are reported in up to 70% of affected patients, most frequently as vesicoureteral reflux (VUR), unilateral renal agenesis, or dysplastic/multicystic kidneys [2]. Advances in reconstructive surgery have improved outcomes, but long-term functional sequelae remain poorly documented [3,4].

Endoscopic treatment of VUR using bulking agents, such as polydimethylsiloxane (Macroplastique^®^, Uroplasty, Inc., Minnetonka, MN, USA) is considered safe and effective. However, its non-resorbable nature has been associated with late complications, including ureteral obstruction, bladder erosion, and calcification [5,6,7,8,9].

We report a rare case of hypotonic, hyporeflexic, large-capacity bladder with bilateral obstructive megaureter in a boy with VACTERL syndrome, highlighting diagnostic and therapeutic challenges.

## 2. Case Presentation

A 16-year-old boy, with a diagnosis at birth of VACTERL syndrome, came to our attention with history of breakthrough febrile urinary tract infections (UTIs), colicky flank pain, and orchiepididymitis. He received multiple surgical interventions during his first years of life to correct esophageal atresia and tracheo-esophageal fistula, Fallot’s tetralogy, lumbosacral lipoma, and tethered cord, which were associated with VACTERL syndrome. Regarding the urogenital system, he had Y-type urethral duplication, with hypospadiac orthotopic urethra and duplicated urethra running from the bladder neck to the anterior anal edge. The accessory urethra was dissected and excised through anterior sagittal trans-ano-rectal approach (ASTRA). Tubularized Incised-Plate Urethroplasty (TIPU) was performed to restore normal meatal position of the hypospadiac orthotopic urethra. Furthermore, he underwent intravesical trans-trigonal Cohen ureteral reimplantation to correct bilateral high-grade VUR at 1 year of age. In the following 2 years, repeated endoscopic subureteric injections with polydimethylsiloxane (Macroplastique^®^) were performed to treat recurrent bilateral VUR. After the ureteral reimplantation and repeated subureteric injections performed during the first three years of life at other centers, the patient did not report urinary tract complications and did not undergo regular urological monitoring until adolescence, when new symptoms appeared. During the year preceding our observation, the patient presented recurrent febrile UTIs and three episodes of orchiepididymitis, all treated medically.

A summary of the patient’s previous surgical procedures and clinical events is presented in Table 1, providing a chronological overview of the major interventions and the onset of symptoms.

At admission, physical examination revealed an intact retractile prepuce and a glandular urethral meatus, with no external signs of previous urethral duplication. However, a weak and prolonged urinary stream requiring abdominal straining was noted. Urine flowmetry (UF) showed an average flow rate of 18.52 mL/s, a voiding time of 61.32 s and a urinary volume of 950 mL. Ultrasonography (US) showed bilateral hydronephrosis with megaureter, a thickened bladder wall, a 2 cm hyperechoic nodule at the left vesicoureteral junction (VUJ), and post-void residual (PVR) > 60% of the bladder capacity. Serum creatinine was 1.4 mg/dL (slightly elevated for age), and Mag-3 renogram showed reduced left split renal function (35.5%) and delayed drainage (T½ > 25 min).

**Figure 1:**
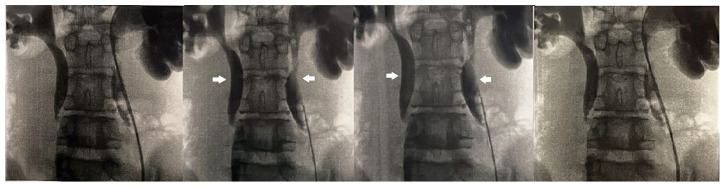
Retrograde pyelography showing bilateral calyceal and ureteral dilatation (white arrows) and delayed contrast washout.

**Figure 2:**
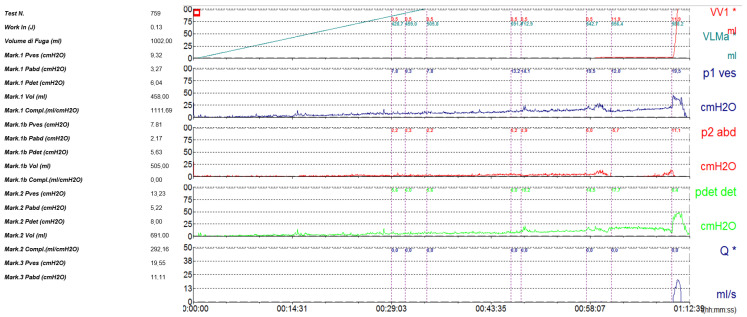
Urodynamic study demonstrating large bladder capacity (1002 mL) and low detrusor pressure (8.4 cm H_2_O), consistent with hypotonic, hyporeflexic bladder.

The anterior urethra presented an adequate caliber at urethrocystoscopy using a pediatric 9.5 F scope (KARL STORZ SE & Co., KG, Tuttlingen, Germany). Hypoplastic *veru montanum* and a blind punctiform orifice, representing the remnant of the resected duplicated urethra, were observed distally to the hypertrophic posterior rim of the bladder neck. The bladder appeared large with slightly trabeculated wall. The right ureteral ostium was narrow and located at the apex of the mound of previous bulking agent implantation, whereas the left ureteral ostium appeared narrow and displaced laterally. After catheterization of both ureteral orifices, retrograde pyelography showed dilatation of both ureters and all calices, which became progressively narrow from the iliac tract to their entry into the bladder, especially on the left side, with delayed contrast washout (Figure 1). Two indwelling 4F ureteral catheters were placed into the left ureter for 8 days to dilate the ureteral orifice. The patient was prescribed urotherapy (alpha-blockers), bladder training with double voiding, and clean intermittent catheterization (CIC) twice a day. No significant improvement was observed at 3-month control, and the patient was poorly compliant with CIC. In the following 8 months, he presented three further episodes of orchiepididymitis, treated with medical therapy. US confirmed persistent bilateral severe hydronephrosis and megaureter, very high bladder capacity (max volume 800 mL), and abnormal PVR (460 mL). To rule out the diagnosis of neurogenic bladder, lumbosacral magnetic resonance imaging (MRI) excluded any intrinsic anomalies of the spinal cord. Urodynamic study revealed a maximum cystometric capacity of 1002 mL, a maximum detrusor pressure of 8.4 cm H_2_O, and a PVR of 350 mL, consistent with hypotonic and hyporeflexic bladder (Figure 2).

After multidisciplinary discussion on the proper urological approach, we planned to correct the bilateral ureteral obstruction and evaluate the outcome on the bladder outlet. We counseled the parents about the possible failure of the planned surgical intervention to restore normal bladder function and the secondary necessity to perform temporary button cystostomy or definitive continent appendicovesicostomy to ensure complete bladder emptying.

**Figure 3:**
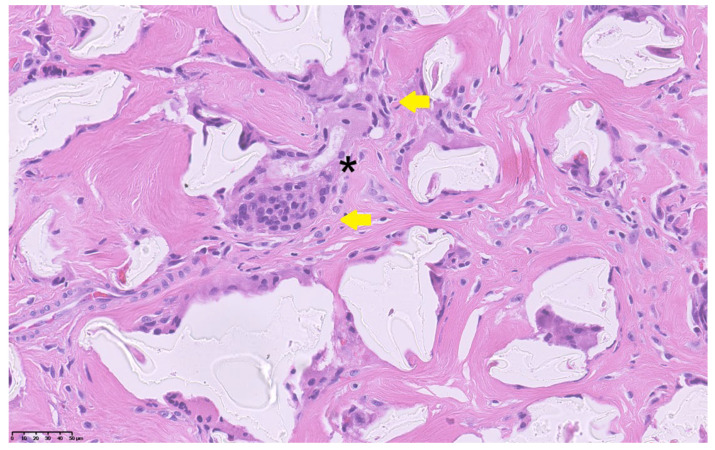
Histopathological section showing foreign-body giant cells (yellow arrows) surrounding amorphous non-resorbable material (asterisk) within a dense fibrotic stroma (H&E stain, 40×). Scale bar = 50 µm.

At surgery, a wide fibrotic reaction was present involving the distal ureters, inter-ureteric band, bladder trigone, and anterior division of the pelvic neurovascular bundle. Both the ureters were carefully dissected to achieve sufficient length and reimplanted longitudinally according to the Politano–Leadbetter technique, after resection of the distal tract. Posterior urethra and bladder neck were calibrated until 26 F, thus excluding any organic stenosis. Limited reductive cystoplasty with fundus invagination was finally performed and a suprapubic catheter was positioned. The pathology report of the excised tissue showed a granulomatous reaction with a predominance of foreign-body giant cells, confirming that the massive fibrotic reaction was caused by the presence of non-resorbable Macroplastique^®^ particles (Figure 3).

**Figure 4:**
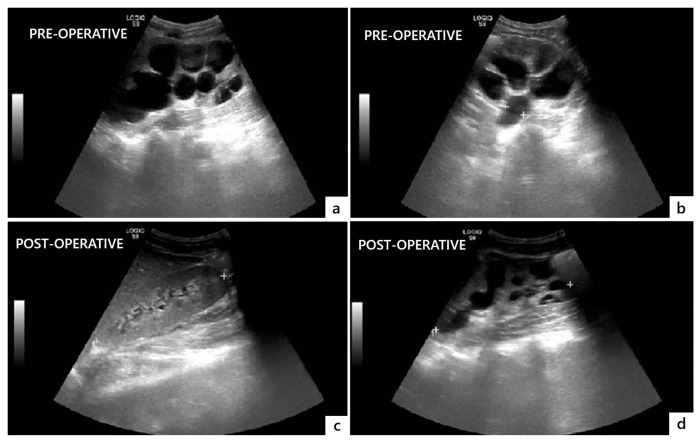
Comparison of preoperative (**a**,**b**) and postoperative (**c**,**d**) ultrasonographic findings showing resolution of bilateral hydronephrosis and megaureter three months after surgery.

The postoperative course was uneventful, and the patient was discharged on postoperative day 14. He was instructed to regularly void at 3 h intervals and measure the PVR through the suprapubic catheter after each micturition. Postoperative US showed a significant decrease in bilateral hydroureteronephrosis and no significant PVR (Figure 4).

After surgery, renal function normalized (creatinine = 0.9 mg/dL), and a renogram demonstrated improved left renal drainage with T½ < 10 min and split renal function = 41%. Uroflowmetry at 3 months showed a maximum flow rate = 22.5 mL/s with a PVR < 50 mL, confirming functional recovery. The suprapubic catheter was removed three months after surgery.

At the three-year follow-up, the patient is asymptomatic and voids regularly with absent or no significant PVR.

## 3. Discussion

This case demonstrates the multifaceted challenges of urological management in patients with VACTERL association [1,2]. The upper and lower urinary tract outcome can be favorable but long-term effects on the bladder function are still awaited [4].

Our patient developed bilateral obstructive megaureter several years after Macroplastique^®^ implantation. The histopathological finding of a foreign-body granulomatous reaction confirmed that the fibrotic process and distal ureteral obstruction were secondary to the persistence of non-resorbable polydimethylsiloxane particles. While the occurrence of late ureteral obstruction and fibrosis after Macroplastique^®^ injection has been largely documented in the literature [5,6,7,8,9], the present case is unique because this complication occurred in a patient with pre-existing urinary tract malformations, thereby complicating both the diagnostic interpretation and the therapeutic approach. The novelty and clinical relevance of the present case does not consist of demonstrating a new causal/eziological link between VACTERL syndrome and Macroplastique^®^-related fibrosis/obstruction, but in documenting how a known late complication of a non-resorbable bulking agent can become diagnostically and therapeutically far more complex when it occurs in a syndromic patient with VACTERL, who already has congenital urinary tract anomalies and multiple prior reconstructions.

The coexistence of a rare long-term complication (Macroplastique^®^-induced fibrosis and bladder dysfunction) in a patient with congenital urological anomalies made the differential diagnosis and surgical decision-making process particularly challenging. At presentation, the patient exhibited a markedly enlarged bladder capacity with elevated post-void residual and bilateral hydronephrosis. These findings could be compatible with either neurogenic bladder, mechanical outlet obstruction, or severe functional decompensation secondary to chronic obstruction. Given his previous history of tethered cord and multiple pelvic surgeries, a neurogenic etiology initially appeared plausible. However, the absence of neurological deficits and normal lumbosacral MRI ruled out a spinal cause. Similarly, cystoscopy and intraoperative calibration excluded any mechanical obstruction at the bladder neck or urethral level. Ischemic or iatrogenic neurovascular injury was considered unlikely, as no previous pelvic procedures involving extensive dissection or devascularization were performed after the urethral surgery. The persistence of bilateral obstruction despite these negative findings prompted consideration of a fibrotic process secondary to previous Macroplastique^®^ injection, an assumption later confirmed by histopathology. This diagnostic pathway, requiring systematic exclusion of several potential etiologies, highlights the importance of maintaining a broad differential diagnosis when evaluating late-onset bladder dysfunction in syndromic patients.

Given the bilateral distal ureteral narrowing and the large, hypocontractile bladder, surgical management required an individualized, stepwise approach. After multidisciplinary discussion, we elected to perform bilateral ureteral reimplantation to relieve obstruction and assess postoperative bladder function before considering any form of diversion. Intraoperatively, the discovery of extensive periureteral and trigonal fibrosis confirmed the suspected iatrogenic mechanism. The addition of a limited reductive cystoplasty was intended to decrease bladder volume, improve detrusor efficiency, and facilitate more effective voiding [10,11]. This combination allowed us to avoid long-term catheterization or continent urinary diversion, restoring spontaneous voiding and preserving renal function.

The histological finding of a granulomatous foreign-body reaction involving the trigonal region and periureteral tissues provides a plausible mechanism linking chronic inflammatory fibrosis with both ureteral obstruction and detrusor underactivity. In this context, the involvement of the anterior neurovascular bundle observed intraoperatively may have contributed to the secondary bladder hypocontractility. Although a direct causal relationship between VACTERL syndrome and the bulking agent complication cannot be demonstrated, the main educational message of this report is that patients with VACTERL often have pre-existing urinary tract malformations and altered pelvic anatomy due to multiple early reconstructions, and therefore a higher chance that a late iatrogenic event presents atypically and is diagnosed late.

The use of non-resorbable bulking agents such as polydimethylsiloxane (Macroplastique^®^) in children is now discouraged because of their demonstrated potential for late complications, including ureteral obstruction, granulomatous inflammation, and calcification [5,6,7,8,9]. In our patient, the endoscopic injections were performed more than 15 years ago, at a time when Macroplastique^®^ was still commonly used for pediatric VUR. Thus, the material choice reflected the clinical practice and therapeutic standards of that time. In contrast, the introduction of dextranomer/hyaluronic acid (Dx/HA) copolymer has significantly reduced the risk of chronic inflammation and obstruction while maintaining high success rates in recurrent VUR management.

Current guidelines recommend Dx/HA as the first-line endoscopic treatment for pediatric VUR due to its biocompatibility and resorbable nature, with reimplantation surgery reserved for failures or complex anatomical cases [12,13]. This case therefore serves as a reminder of the evolution of endoscopic management standards and the need for careful counseling and informed consent in the use of any bulking agent in children [14,15]. Beyond endoscopic treatment, alternative management options for recurrent VUR include long-term antibiotic prophylaxis for low-grade reflux, laparoscopic or robotic ureteral reimplantation for high-grade or persistent cases, and temporary urinary diversion in patients with severe bladder dysfunction or infection risk. The choice of therapy should be individualized according to the grade of reflux, bladder dynamics, renal function, and previous interventions [16,17,18].

Lastly, this case underscores several key clinical lessons. First, a thorough and multidisciplinary diagnostic assessment is essential to differentiate mechanical, neurogenic, and iatrogenic causes of bladder dysfunction, particularly in syndromic patients with prior reconstructions. Second, long-term renal and bladder surveillance is mandatory in all patients who have undergone endoscopic correction of VUR with non-resorbable agents, even many years after treatment, since complications may manifest decades later. Third, the case illustrates the value of individualized surgical strategies combining ureteral reimplantation with reductive cystoplasty to reestablish functional voiding in selected large-capacity bladders. Finally, this experience reinforces a broader principle for current pediatric urological practice: careful selection of biocompatible, resorbable materials and transparent counseling of families about potential long-term sequelae are fundamental to ensuring patient safety and informed decision-making.

## 4. Conclusions

This case underlines the diagnostic and therapeutic challenges of managing late urological complications in a VACTERL patient with pre-existing urinary anomalies. The overlap of congenital and iatrogenic factors made the diagnostic pathway complex, requiring careful exclusion of neurogenic and mechanical causes. A tailored surgical strategy combining ureteral reimplantation and limited reductive cystoplasty restored bladder function and preserved renal outcome. This experience underscores the need for multidisciplinary evaluation, cautious material selection, and long-term urological follow-up in patients treated with non-resorbable bulking agents.

## Figures and Tables

**Table 1:** Chronological summary of main urological and surgical events.

Age (Years)	Procedure/Event	Institution/Note	Outcome
0	Diagnosis of VACTERL association	—	—
0–1	Repair of esophageal atresia and tracheoesophageal fistula	External center	Uneventful recovery
0–1	Correction of Fallot’s tetralogy	External center	Good cardiac function
1	Bilateral Cohen ureteral reimplantation for high-grade VUR	External center	Uneventful recovery
1–2	Surgical removal of lumbosacral lipoma and detethering of spinal cord	External center	No neurological sequelae; normal postoperative MRI
1–3	Two endoscopic subureteric injections of Macroplastique^®^ for recurrent bilateral VUR	External center	Temporary improvement reported
2	Excision of accessory urethra (Y-type duplication) and TIP urethroplasty	External center	Normal orthotopic meatus; good healing
3–15	No urological follow-up; asymptomatic period	—	—
15–16	Recurrent febrile UTIs and three episodes of orchiepididymitis	Before referral	Treated medically

## Data Availability

The data published in this study are available upon request from the corresponding author.
